# The MacroH2A1.1 – PARP1 Axis at the Intersection Between Stress Response and Metabolism

**DOI:** 10.3389/fgene.2018.00417

**Published:** 2018-10-09

**Authors:** Sarah Hurtado-Bagès, Iva Guberovic, Marcus Buschbeck

**Affiliations:** ^1^Josep Carreras Leukaemia Research Institute, Campus ICO-Germans Trias i Pujol, Universitat Autònoma de Barcelona, Badalona, Spain; ^2^Ph.D. Program in Biomedicine, Department of Experimental and Health Sciences, Universitat Pompeu Fabra, Barcelona, Spain; ^3^Ph.D. Program in Biomedicine, Faculty of Pharmacy and Food Science, University of Barcelona, Barcelona, Spain; ^4^Program for Predictive and Personalized Medicine of Cancer, Germans Trias i Pujol Research Institute (PMPPC-IGTP), Badalona, Spain

**Keywords:** epigenetic, metabolism, stress response, macroH2A1.1, PARP1

## Abstract

The exchange of replication-coupled canonical histones by histone variants endows chromatin with specific features. The replacement of the canonical H2A histone for the histone variant macroH2A is one of the most remarkable epigenetic modifications. The three vertebrate macroH2A proteins have a unique tripartite structure consisting of H2A-like domain, unstructured linker, and macrodomain. Macrodomains are ancient globular folds that are able to bind nicotinamide adenine dinucleotide (NAD^+^) derived metabolites. Here, we will briefly describe the physiological relevance of the metabolite binding in the context of chromatin. In particular, we will focus on the macroH2A1.1 isoform that binds ADP-ribose and poly-ADP-ribose polymerase 1 (PARP1) enzyme, a cellular stress sensor. We will discuss the impact of this interaction in the context of cancer, senescence, cell stress and energy metabolism.

## Introduction

The adaptation of cells and organisms to the environment requires coordinate and rapid response to external stimuli and stress. In order to do so, throughout evolution cells developed complex molecular mechanisms that directly influence gene regulation without changing the DNA sequence. These mechanisms, also called epigenetic regulation, modify the chromatin composition and structure. The exchange of replication-coupled histones for histone variants is one of the major chromatin modifications. Histone variants differ from replication-coupled canonical histones in their protein structure, timing of expression, and genomic distribution (Buschbeck and Hake, 2017). Depending on the cellular context, they are incorporated into chromatin in a locus-specific manner by specialized protein chaperones and ATP-dependent chromatin remodeling enzymes. In this way, histone variant incorporation endows chromatin with particular properties. During the last years, it has become clear that histone variants play key roles in epigenetically regulated processes such as development, cancer, and somatic cell reprogramming (Buschbeck and Hake, 2017). Within a diverse group of H2A variants, macroH2As are the most divergent group of histone variants with a unique tripartite structure (Buschbeck and di Croce, 2010). The hallmark of macroH2A structure is its evolutionary conserved C-terminal macrodomain. Intriguingly, this macrodomain mediates the interaction of the macroH2A1.1 isoform with poly-ADP-ribose polymerase 1 (PARP1) enzyme, which is a nuclear stress sensor (Kustatscher et al., 2005). Here, we will discuss the effect of this interaction on cell physiology with a particular focus on stress response and cell metabolism. We believe that a better understanding of the macroH2A1.1-PARP1 axis will help to interpret reported loss- and gain-of-function phenotypes.

## Presenting the Players

### MacroH2A1.1 – A Structural Chromatin Component Able to Bind ADP-Ribose

MacroH2A histone variants are highly conserved since the emergence of multicellular life (Rivera-Casas et al., 2016). They are widely distributed across the genome and make up approximately 1% of the total H2A pool (Buschbeck and Hake, 2017). In mammals, two genes, H2AFY and H2AFY2, encode for macroH2A1 and macroH2A2, respectively (Pehrson and Fried, 1992; Costanzi and Pehrson, 2001). Alternative splicing of macroH2A1 further gives rise to macroH2A1.1 and macroH2A1.2 isoforms (Pehrson et al., 1997). MacroH2As are unique for their atypical tripartite protein structure comprised of an N-terminal H2A-like domain, which is fused by an unstructured linker region to a large C-terminal macrodomain (Chakravarthy et al., 2005; Kustatscher et al., 2005). It was recently demonstrated that macroH2A plays a major structural role in the maintenance of heterochromatin architecture and nuclear organization (Fu et al., 2015; Douet et al., 2017). While macroH2A has a role in transcriptional repression (Costanzi and Pehrson, 1998; Mermoud et al., 1999), it is also involved in signal-induced gene activation (discussed in Creppe et al., 2012) and in suppressing transcriptional noise (Lavigne et al., 2015). In this way, macroH2A directly or indirectly affects transcriptional regulation in an ambivalent manner.

Loss-of-function studies have shown that macroH2A promotes differentiation of embryonic and adult stem cells (Pasque et al., 2012) and inhibits somatic cell reprogramming (Pasque et al., 2011). While macroH2As contribute to the robustness of embryonic development in zebrafish (Buschbeck et al., 2009), mice lacking a single macroH2A gene develop without overt developmental defects and are viable and fertile (Changolkar et al., 2007; Boulard et al., 2010). On the other hand, mice lacking both macroH2A-encoding genes are growth retarded (Pehrson et al., 2014). In cancer, macroH2A1.1 and macroH2A2 mostly act as tumor suppressors, while the role of macroH2A1.2 seems to be largely context- and cell type- dependent (Cantariño et al., 2013; Corujo and Buschbeck, 2018).

It remains unclear if the macrodomain of macroH2A is involved in the general function of the protein. Crystal structures of macroH2A macrodomains revealed the presence of a binding pocket within all isoforms. Nevertheless, these binding pockets differ significantly in size and hydrophobicity (Kustatscher et al., 2005). Generally, macrodomains can be found across most species (Rack et al., 2016). They are ancient globular domains that are able to bind ADP-ribose moieties and derivatives (reviewed in Posavec et al., 2013). Ladurner group made a pioneering discovery showing that macroH2A1.1 macrodomain also specifically binds nicotinamide adenine dinucleotide (NAD^+^) derived metabolites such as ADP-ribose and *O*-acetyl-ADP-ribose (Kustatscher et al., 2005). This binding of ADP-ribose enables macroH2A1.1 to interact with activated PARP1 enzyme (Karras et al., 2005; Timinszky et al., 2009). On the other hand, macroH2A1.2 and macroH2A2 isoforms are unable to bind these metabolites.

### PARP1 – A Major Nuclear Stress Sensor of the Cell

Poly-ADP-ribose polymerases, also known as diphteria toxin-like ADP-ribosyltransferases (ARTDs), are a conserved superfamily of enzymes present in all domains of life, from bacteria to human (Citarelli et al., 2010; Daugherty et al., 2014). In mammals, up to 17 PARP genes share specific signature motifs in the catalytic domain (Luo and Kraus, 2012). PARPs are stress sensor enzymes that are best studied in the context of DNA damage repair (reviewed in Amours et al., 1999), transcription, and chromatin organization (reviewed in Kraus and Lis, 2003). On the whole-cell level, PARPs regulate several cellular processes including proliferation and differentiation, apoptosis, pro-inflammatory responses, neuronal long-term memory, mitochondrial function, and metabolic stress (for reviews, please see Chiarugi, 2002; Schreiber et al., 2006; Languren et al., 2013; Vida et al., 2017).

Poly-ADP-ribose polymerase 1 is the founding and the best characterized member of the PARP superfamily (Ame et al., 1999). It is highly abundant in eukaryote nuclei and one of three nuclear PARPs that are activated by discontinuous DNA structures caused by DNA damage. Indeed, PARP1 is implicated in the repair of single-strand or double-strand breaks either by homologous recombination or non-homologous end joining (reviewed in Rouleau et al., 2010). When activated, PARP1 hydrolyzes NAD^+^ generating ADP-ribose moieties (reviewed in Schreiber et al., 2006). Consequently, PARP1 transfers this newly formed ADP-ribose to target proteins resulting in mono- or poly-ADP-ribosylation (MARylation and PARylation, respectively). The main targets of activated PARP1 are histones and PARP1 itself, as a consequence of auto-PARylation (reviewed in Kim et al., 2005).

Poly-ADP-ribose polymerase 1 is composed of three functional domains, namely, N-terminal DNA-binding domain, central auto-modification domain, and C-terminal catalytic domain (Langelier et al., 2012). The binding to damaged DNA mediates conformational change of PARP1 increasing its affinity for NAD^+^ and leading to the activation of PARP1 catalytic domain. PAR formation has at least three functions: (i) creation of a platform for the recruitment of effector proteins including the DNA repair machinery, (ii) disruption of protein-protein or protein-nucleic acid interactions leading to chromatin relaxation, and (iii) generation of signal for protein ubiquitination and degradation (reviewed in Gibson and Kraus, 2012). Auto-PARylation of PARP1 reduces its affinity toward DNA creating a negative feedback loop which inhibits its own activity (Zahradka and Ebisuzaki, 1982). Removal of PARP1 from DNA and rapid enzymatic turnover of PAR are essential for optimal function of the DNA repair machinery (reviewed in Rouleau et al., 2010). Interestingly, it has been reported that PARP1 can also be activated in a DNA-independent manner through the interaction with ERK2 (extracellular signal-regulated kinase 2) (Cohen-Armon et al., 2007). Therefore, this may implicate PARP enzymes in other cellular processes unrelated to DNA, opening new lines of research in the field of PARP.

*In vivo* studies showed that mice lacking PARP1 are hypersensitive to genotoxic agents or γ-irradiation, demonstrating the importance of PARP1 for efficient DNA repair (Ménissier-de Murcia, 1997; Wang et al., 1997). Pharmacological inhibition of PARP1 was first introduced in cancer therapy to exploit synthetic lethality of BRCA1-deficient breast tumors (Fong et al., 2009) and is now widely tested in combination with DNA damaging chemotherapeutic drugs (Farmer et al., 2005). In addition to cancer therapy, PARP inhibitors may have additional benefits in other diseases such as cardiovascular or metabolic disorders (Pacher and Szabo, 2007; Shevalye et al., 2010).

### Physiological Studies Link MacroH2A1 and PARP1 to Metabolism

Systemic loss-of-function studies in mice linked both macroH2A1 and PARP1 to metabolic phenotypes, although some observations are controversial and sometimes lead to opposite conclusions. Here, we summarize and discuss some of these findings (for a more comprehensive view, please see **Table 1**).

**Table 1:** Impact of macroH2A1.1-PARP1 axis on metabolic phenotypes.

		Model system			Metabolic phenotype			Reference
		Mice strain or cell line	Gender	Age (weeks)	Experimental manipulation	Description	Outcome	
MacroH2A1	Loss or gain of function	C57BL/6 mice	Mainly Male	From 1 to 8	MacroH2A1 KO	Fertile and viable, develop glucose intolerance. Deregulation of genes involved in liver metabolism during new born-adult transition.	Negative	Changolkar et al., 2007
		129Vx C57BL/6 mice	Female	From 6 to 12	MacroH2A1 KO	50% exacerbated liver steatosis.	Negative	Boulard et al., 2010
		129Vx C57BL/6 mice	Mainly Male	From 1 to 8	MacroH2A1 double KO	Smaller with reduced lean body mass. More susceptible to perinatal death, reproductive, and mothering troubles. Deregulation of genes involved in liver metabolism.	Negative	Pehrson et al., 2014
		129Vx C57BL/6 mice	Male	From 12 to 24	MacroH2A1 KO	Fat mass reduction and protected against HFD-induced obesity. Up-regulation of thermogenic genes, downregulation of adipogenic genes. Enhance glucose tolerance, increase of total activity during nights.	Positive	Sheedfar et al., 2015
		C57BL/6	Male	From 6 to 18	MacroH2A1.2 gain of function	Increased leaness, glucose tolerance, decrease fat accumulation in the liver and pancreas.	Positive	Pazienza et al., 2016
		human HepG2 hepatocytes cell line	Male-derived	/	MacroH2A1.2 overexpression	Activation of lipogenic genes, enhanced lipid uptake, and triglycerides.	Negative	Podrini et al., 2014
		human HepG2 hepatocytes cell line	Male-derived	/	MacroH2A1.1 overexpression	Protected against lipid accumulation with reduction of triglycerides.	Positive	
		human HepG2 and murine Hepa1-6 cell line	Male-derived	/	MacroH2A1.1 overexpression	Protected hepatocytes against lipid accumulation.	Positive	Pazienza et al., 2014
		murine 3T3-L1 adipocytes cell line	Male-derived	/	MacroH2A1.1 downregulation (shRNAs)	Adipogenesis abrogation via EZH2 interaction and regulation of Wnt signaling.	Positive	Wan et al., 2017
		murine C2C12 myotubes cell line	Female-derived	/	MacroH2A1.1 downregulation (siRNAs)	Altered mitochondrial activity, mitochondrial NAD^+^ and NMN depletion.	Negative	Posavec Marjanović et al., 2017

PARP1	Loss or gain of function	C57BL/6 mice	Male	From 0 to 46	PARP1 KO	Lower body weight gain with HFD. Increased energy expenditure and higher glucose tolerance. Better glucose clearance and decrease of FFA and Triglycerides.	Positive	Bai et al., 2011; Erener et al., 2012a
		C57BL/6 mice	Male	From 0 to 13	PARP1 KO	Increased of hepatic steatosis.	Negative	Erener et al., 2012a
		C57BL/6 mice	Male	From 4 to 46	PARP1 KO	Increased of mitochondrial biogenesis and NAD^+^ content via SIRT1 activity.	Positive	Bai et al., 2011
		C57BL/6 mice	Not indicated	Not indicated	PARP1 KO	Altered circadian rhythms and food intake.	Negative	Asher et al., 2010,
		C57B1/6 mice	Not indicated	Not indicated	Ectopic integration of human PARP1	Premature development of inflammation and age-associated pathologies. Gain weight and glucose intolerance.	Negative	Mangerich et al., 2010

PARP1	Loss or gain of function	129/SvlmJ mice 129VxC57BL/6 mice	Mainly male	From 4 to 19	PARP1 KO	Gain weight; and increased susceptibility to high-fat diet-induced obesity. Hyperglycemia, hyperinsulinemia, increase of plasma leptin levels.	Negative	Wang et al., 1997; Devalaraja-Narashimha and Padanilam, 2010
						Increase of fat mass and decrease of lean mass.		
		129Vx C57BL/6 mice	Not indicated	From 4 to 10	PARP1 KO	Protection against streptozotocin diabetes.	Positive	Burkart et al., 1999
		129Vx C57BL/6 mice	Not indicated	20	PARP1 KO	Protection against *Trypanpsoma cruzi*-induced cardiomyopathy. Improve mtDNA content, mitochondrial activity and oxidative stress.	Positive	Wen et al., 2018
		mice (unclear from Jackson laboratory)	Male	From 4 to 48	PARP1 KO	Prevented drastic decrease of β-cell proliferation during aging.	Positive	Gong et al., 2012
		human HEK293T kidney cells	Female-derived	/	PARP1 KO	Enhanced mitochondrial gene expression and respiration.	Positive	Bai et al., 2011;
		murine 3T3-L1 adipocytes	Male-derived	/	PARP1 KO	Reduced adipocyte differentiation and PPARy-dependent gene expression.	Positive	Erener et al., 2012b; Lehmann et al., 2015
		murine C2C12	Male-derived	/	PARP1 KO	Increase of adipogenic transcriptional program through C/EBPβ PARylation.	Negative	Luo et al., 2017
		murine C2C12 myotubes cell line	Female-derived	/	PARP1 siRNA	Increased oxidative phosphorylation and glycolytic activity.	Positive	Oláh et al., 2015
PARP1	Inhibition	C57BL/6 mice	Male	From 4 to 46	Inhibition (PJ34)	Better lipid metabolic profile, increase of NAD^+^ level in Brown adipose tissue and skeletal muscle.	Positive	Bai et al., 2011
		C57BL/6 mice	Not indicated	20	Inhibition (PJ34)	Enhanced mitochondrial biogenesis in Chagas mice with myocardial fibrosis.	Positive	Wen et al., 2018
		C57BL/6 mice	Male	From 10 to 28	Inhibition (MRL-45696)	Enhanced mitochondrial content and activity in Brown adipose tissue and skeletal muscle.	Positive	Pirinen et al., 2014
		129Vx C57BL/6 mice	Not indicated	Not indicated	Inhibition (nicotinamide)	Prevented pancreatic β-cell destruction.	Positive	Burkart et al., 1999
		Wistar rat	Male	Not indicated	Inhibition (nicotinamide, or 3-ABA)	Improved β-cell regeneration and prevents diabetes mellitus.	Positive	Yonemura et al., 1984
		diabetic db/db(Leprdb/db) mice	Male	5	Inhibition (lNO1001; PJ34)	Prevented diabetes induces podocytes and blocks their hyperglycemia.	Positive	Szabó et al., 2006
		murine C2C12 myotubes cell line	Female-derived	/	Inhibition (PJ34)	Better mitochondrial activity and enhanced oxidative metabolism.	Positive	Bai et al., 2011; Posavec Marjanović et al., 2017

*The table summarizes the different metabolic phenotypes observed when PARP1 or macroH2A1s are modulated *in vivo* and *in vitro*. Positive or negative outcomes from such manipulation are shown in the outcome section*.

Although mice lacking macroH2As display only mild phenotypes, they repeatedly exhibit metabolic disorders. Indeed, macroH2A1 knockout (KO) mice displayed impaired clearance of bolus glucose injections, suggesting a pre-diabetic phenotype and partial insulin resistance (Changolkar et al., 2007). However, when fed with high-fat diet, increased energy expenditure was observed and was accompanied by lower accumulation of fat and increased leanness (Sheedfar et al., 2015). Furthermore, deregulation of genes involved in lipid metabolism was reported in the liver of mice from different genetic backgrounds (Changolkar et al., 2007; Boulard et al., 2010). However, liver fat accumulation was only observed in female mice with 50% penetrance (Boulard et al., 2010). Liver cancer cell lines showed to be protected from lipid accumulation upon overexpression of macroH2A1.1 (Pazienza et al., 2014). Moreover, overexpression of macroH2A1.2 in mice was found to reduce adipose tissue and thus to promote leanness (Pazienza et al., 2016). In summary, macroH2A has been reported as a metabolic regulator with opposing effects in mice. Such discrepancies might be explained by the use of mice with different genetic backgrounds, ages or genders and distinct outcomes can be observed depending on the macroH2A isoforms depleted (**Table 1**).

Similarly to macroH2A1, contradictory results have been observed with PARP1 KO mice, displaying improved or deteriorated metabolic fitness (reviewed in Bai and Cantó, 2012; **Table 1**). On the one hand, mice lacking PARP1 displayed aberrant circadian rhythms (Asher et al., 2010) and diet-induced obesity (Devalaraja-Narashimha and Padanilam, 2010) associated with hepatic steatosis (Erener et al., 2012a). On the other hand, PARP1 inhibition and deletion in mice were associated with improvement of diabetes- or obesity-related parameters such as glucose tolerance, weight loss, or fat mass reduction (reviewed in Bai and Cantó, 2012; **Table 1**). Furthermore, PARP1 depletion is correlated with improved mitochondrial and heart function (Wen et al., 2018). Plethora of studies showed that PARP1 can contribute to both gene activation and repression. For example, its interaction with metabolic regulatory transcription factors, such as FOXO1 (Sakamaki et al., 2009) and PPARγ (Erener et al., 2012a), affects genes implicated in mitochondrial respiration and fatty acid oxidation (Bai et al., 2011).

In a living system, different tissues can affect each other’s homeostasis and therefore give rise to differential physiological phenotypes. This could explain the conflicting results observed in macroH2A1 and PARP1 KO mice. Despite discrepancies, the majority of studies suggest unfavorable effects of PARP1 enzyme on metabolism. On the other hand, macroH2A1.1 is often associated with beneficial metabolic outcomes (**Table 1**).

## The macroH2A1.1-PARP1 Axis

Although macroH2A and PARP proteins have been studied for decades, the physiological consequences and mechanisms of their interaction are still unclear and require further research. Depending on the relative abundance of the two proteins and the strength of PARP1 activity, their interaction may have at least two different outcomes. On the one hand, macroH2A1.1 can recruit active PARP1 to chromatin and thus act in concert to regulate gene expression. On the other hand, macroH2A1.1 can bind and inhibit PARP1 activity, with global consequences on cellular metabolism. Although an interconnection between macroH2A1.1 and PARP1 was also observed in the context of DNA damage response (Timinszky et al., 2009), the molecular mechanism of such processes needs to be further investigated.

### The Effect on Gene Regulation

**FIGURE 1:**
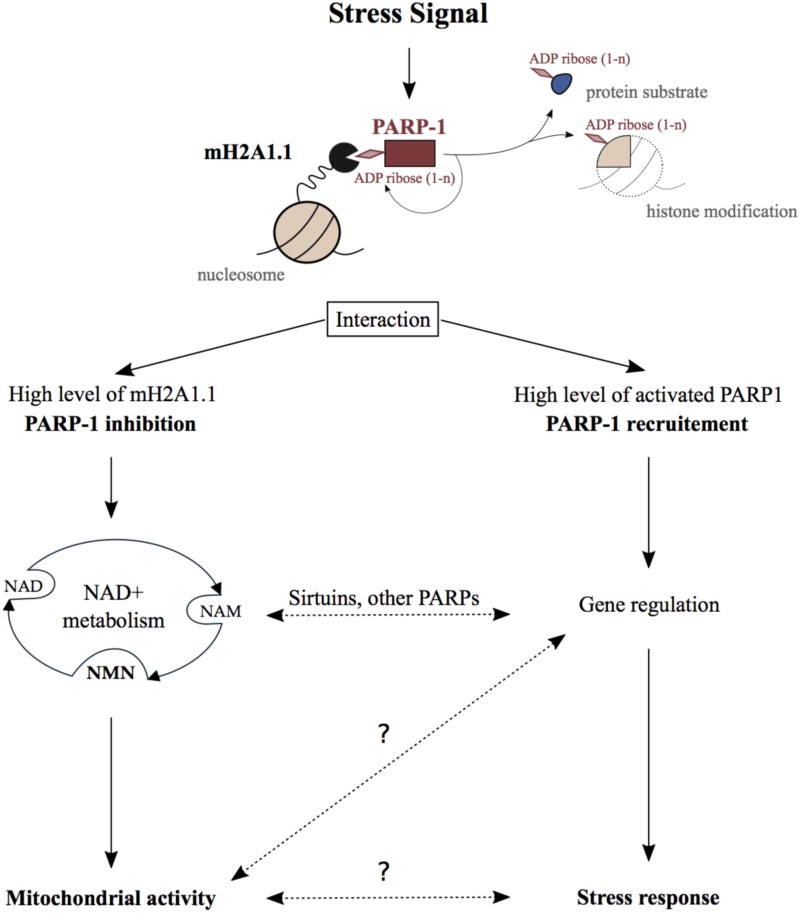
Physiological function of the MacroH2A1.1_PARP1 axis. Stressing signals generated during DNA damage repair, senescence, hormonal response, heat shock, or differentiation promote the binding of macroH2A1.1 to activated PARP1 creating the macroH2A1.1-PARP1 axis. On the one hand, when macroH2A1.1 is highly expressed, this interaction leads to the inhibition of PARP1 activity which reduces PARP1 consumption of nuclear NAD+. In this way, the macroH2A1.1-PARP1 axis influences mitochondrial activity through subcellular NAD pools turnover. On the other hand, macroH2A1.1 and active PARP1 act in concert in order to regulate gene expression and as a consequence regulate stress signals response. Crosstalks between those both pathways implicating sirtuin and other PARP enzymes may additionally influence the physiological outcomes of the macroH2A1.1-PARP1 axis. The question marks illustrate the lack of knowledge regarding some steps in this axis pathway.

MacroH2A1.1 and PARP1 cooperate to repress and activate genes in response to external signals. This is best understood in the context of the transcriptional regulation of the heat shock-inducible *Hsp70.1* gene. In HeLa cells, exogenous macroH2A1.1 was found to recruit PARP1 to the silenced *Hsp70.1* promoter (Ouararhni et al., 2006). Heat shock activates PARP1 resulting in significant auto-PARylation and PARylation of target proteins. This leads to the release of both macroH2A1.1 and PARP-1 from chromatin, and as consequence activates the *Hsp70.1* gene (Ouararhni et al., 2006). In fibroblasts, the interaction of macroH2A1.1 with activated PARP1 contributes to both positive and negative gene regulation (Chen et al., 2014). Mechanistically, macroH2A1.1-bound PARP1 recruits the histone acetyltransferase CBP promoting H2B acetylation of its target genes (Chen et al., 2014). Furthermore, macroH2A1.1-PARP1 axis is important for the regulation of SASP (senescence-associated secretory phenotype) genes in cancer cell model (Chen et al., 2015). Additionally, macroH2A1.1 facilitates differentiation of 3T3-L1 preadipocytes by inhibiting regulatory genes such as *Wnt10b* (Wan et al., 2017). This phenotype was only observed with macroH2A1.1 isoform suggesting the potential implication of macroH2A1.1-PARP1 axis in adipogenesis. In summary, macroH2A1.1-PARP1 axis regulates cellular stress response in a transcription-dependent manner (**Figure 1**). Consequently, it affects several physiological outcomes such as cancer, senescence, and possibly adipogenesis.

### The Effect on NAD^+^ Metabolism

Beside gene regulation, the macroH2A1.1-PARP1 axis was reported to influence cellular NAD^+^ pools. NAD^+^ is a well-known coenzyme essential for redox reactions in metabolism. Beyond its crucial role in glycolysis and mitochondrial respiration, NAD^+^ is also involved in the regulation of gene expression, DNA repair, calcium signaling, circadian rhythms, lifespan, and cell death (reviewed in Cantó et al., 2015). The maintenance of NAD^+^ levels is mainly ensured through its salvage which requires much fewer enzymatic reactions than its de novo synthesis from dietary sources (reviewed in Verdin, 2015). The consumption of NAD^+^ by PARPs and sirtuin deacetylases (SIRTs) generates ADP-ribose and NAM (nicotinamide). NAM is subsequently converted into nicotinamide mononucleotide (NMN), which is a major NAD^+^ precursor (**Figure 1**). Interestingly, more than 50 years ago, it was shown that NMN addition to liver nuclear extract stimulates PARylation supporting the link between NAD^+^ salvage pathway and PARP1 activity (Chambon et al., 1963). Since then, NAD^+^ and its salvage pathway have been demonstrated to be essential for nuclear, cytoplasmic, and mitochondrial activities.

A breakthrough appeared with the development of molecular tools able to detect free NAD^+^ (Cambronne et al., 2016) and ATP (Imamura et al., 2009) in sub-cellular compartments. This allowed for monitoring of how the imbalance of NAD^+^ and ATP in one organelle affects the whole cellular metabolism (Wright et al., 2016; Ryu et al., 2018). Among all PARPs, PARP1 is the major NAD^+^ consumer in the nucleus (reviewed in Fouquerel and Sobol, 2014). Prolonged PARP1 activation leads to depletion of cellular NAD^+^ and ATP (Hassa et al., 2006). As a consequence, this leads to a misbalance of sub-cellular NAD^+^ pools, which results in mitochondrial dysfunction (Virág et al., 1998a; Cipriani et al., 2005), increased oxidative stress and cell death (Virág et al., 1998b; Zong et al., 2004).

Proliferative cells have high nuclear energy demand and mainly use glycolysis to ensure fast synthesis of nucleic acids. On the other hand, terminally differentiated cells rather rely on mitochondrial respiration in order to maintain tissue functions (Leary et al., 1998). In this respect, in commonly used cell model for skeletal muscle differentiation, it was shown that the macroH2A1.1-PARP1 axis protected differentiated myotubes from oxidative stress (Oláh et al., 2015; Posavec Marjanović et al., 2017). Indeed, a decrease of PARP1 protein level and activity was observed during muscle differentiation which was associated with higher resistance to oxidative damage (Bai et al., 2011; Szczesny et al., 2013; Oláh et al., 2015). Furthermore, a splicing switch of macroH2A1 occurs during cell differentiation, which results in the increase of macroH2A1.1 levels (Sporn and Jung, 2012; Posavec Marjanović et al., 2017). Additionally, our group observed that high abundance of macroH2A1.1 leads to the reduction of nuclear consumption of NAD^+^ by PARP1 in differentiated myotubes. In this way, macroH2A1.1 maintains optimal NAD^+^ and NMN levels in the different cellular compartments, thereby facilitating proper respiratory capacity of mitochondria (Posavec Marjanović et al., 2017). Interestingly, dietary supplementation with NMN enhanced mitochondrial oxidative metabolism in mice which was associated with the suppression of age-associated body weight (Yeung et al., 2016). Following a similar trend, pharmacologic inhibition of PARP1 or its genetic deletion improves mitochondrial function (Pirinen et al., 2014). This happens due to the increase of NAD^+^ availability, which promotes SIRT1 activation and downstream regulation of metabolic genes (Bai et al., 2011). In conclusion, the macroH2A1.1-PARP1 axis can influence NAD^+^ metabolism and consequentially the activity of distant organelles, such as mitochondria (**Figure 1**). This can occur independently of gene regulation or could involve other NAD^+^ sensitive transcriptional processes.

## Conclusion and Future Perspectives

MacroH2As are commonly studied with respect to their function as transcriptional regulators and epigenome stabilizers. Intriguingly, recent results highlighted a new function of macroH2A1.1, which is to establish adequate threshold for PARP1 activity and help maintain cell energy homeostasis. Future work will be necessary to better understand how different stress signals affect the macroH2A1.1-PARP1 axis and a possible involvement of other partners, such as SIRTs or other PARPs. This new discovery redefines research boundaries of macroH2A field and raises the need to investigate the function of macroH2A considering its effects on both nuclear and whole-cell level. In conclusion, the study of the macroH2A1.1-PARP1 axis could have a great clinical relevance, especially in diseases related to NAD^+^ deficiency, such as sarcopenia and pellagra.

## Author Contributions

SH-B, IG, and MB wrote this review.

## Conflict of Interest Statement

The authors declare that the research was conducted in the absence of any commercial or financial relationships that could be construed as a potential conflict of interest.
